# Supplementary material for: Structural Characterization of the Metalized Radical Cations of Adenosine ([Ade+Li-H]•+ and [Ade+Na-H]•+) by Infrared Multiphoton Dissociation Spectroscopy and Theoretical Studies
Source: Int J Mol Sci. 2023 Oct 20;24(20):15385. doi: 10.3390/ijms242015385 (PMC10607295; doi:10.3390/ijms242015385)
Supplement: Supplementary file 1 [file ijms-24-15385-s001.zip › ijms-2630092-supplementary.pdf]

International Journal of
Molecular Sciences

MDPI

Supporting information

Table of contents

Figure 2

Figure S1. Mass spectra and tandem mass spectra of [IAde+M]+, M = Li, Na). 2

Figure S2. Minimum energy structures of [Ade+K-H]•+ with diffrerent coordination sites. The calculation are performed at the B3LYP-D3/6-311+G(d,p) level. 3

Scheme 4

Scheme S1. Eight coordination situations for the alkali metal cations to interact with 2-I-adenosine... 4

Table S1 Vibrational mode assignment of [Ade+Li/Na-H]•+ **Error! Bookmark not defined.**

Cartesian coordinates of some structures 5

Li-N3-O′-O5′-C2H-1 5

Li-N3-O′-O5′-C2H-2 6

Li-N3-O′-O5′-C2H-3 7

Na-N3-O′-O5′-C2H-1 8

Na-N3-O′-O5′-C2H-2 9

Na-N3-O′-C2H-1 10

International Journal of
Molecular Sciences

Figure

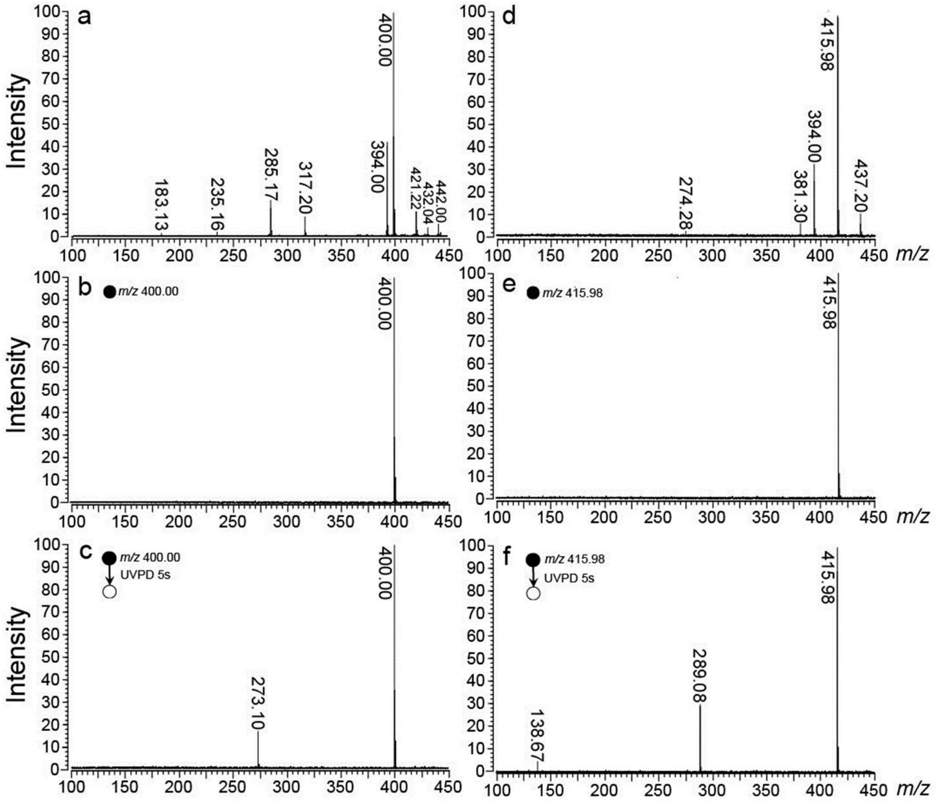

Figure S1. Mass spectra and tandem mass spectra of [^{I}Ade+M]$^{+}$, M = Li, Na. Among them, (a), (b) and (c) are for [^{I}Ade+Li]$^{+}$, and (d), (e) and (f) are for [^{I}Ade+Na]$^{+}$. They are the mass spectra of metalized cations of 2-I-adenosine before (a) and (d) and after (b) and (e) isolation. And (c) and (f) are their tandem UVPD mass spectra of the precursor ions. The wavelength of the UV laser applied here is 280 nm, and the irradiation periods are 5 seconds.

International Journal of *Molecular Sciences*

MDPI

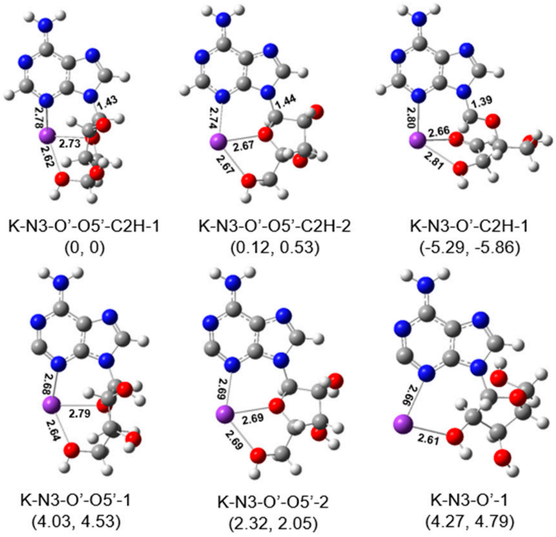

Figure S2. Minimum energy structures of $[Ade+K-H]^{\bullet+}$ with different coordination sites. The calculations were performed at the B3LYP-D3/6-311+G(d,p) level.

Table

Table S1 Vibrational mode assignment of $[Ade+Li/Na-H]^{\bullet+}$.

$[Ade+Li-H]^{\bullet+}$			$[Ade+Na-H]^{\bullet+}$		
Exp.	Cal.[a]	Vibrational mode	Exp.	Cal. [b]	Vibrational mode
3460	3450	NH_2 symmetric vibration	3468	3457	NH_2 symmetric vibration
3580	3578	NH_2 asymmetric vibration	3580	3575	NH_2 asymmetric vibration
3635	3622	H-bonded OH stretch	---	---	---
3710	3705	Free OH stretch	3710	3705	Free OH stretch

[a]Ref to the isomer of Li-N3-O'-O5'-C2H-2.

[b]Ref to the isomer of Na-N3-O'-C2H-1.

International Journal of
Molecular Sciences

Scheme

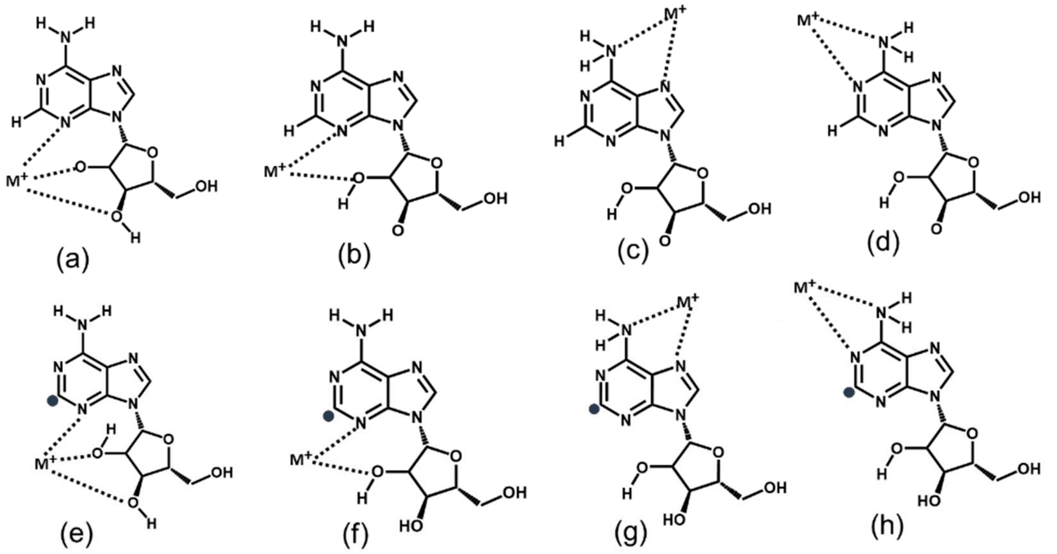

Scheme S1. Eight coordination situations for the alkali metal cations to interact with 2-I-adenosine.

International Journal of
Molecular Sciences

Cartesian coordinates of some structures

Li-N3-O′-O5′-C2H-1

C	2.21119400	1.99773500	0.60490900
N	3.45269000	1.54995100	0.61402900
C	3.71060700	0.29084600	0.18892700
C	2.62287200	-0.50424200	-0.23369800
N	1.09355000	1.34034100	0.21474800
C	1.36602400	0.08173100	-0.17893400
N	4.97159500	-0.14452100	0.18938000
H	5.19281300	-1.07979000	-0.11522400
H	5.70655400	0.46684600	0.51105600
N	2.54499700	-1.80215600	-0.70727000
C	1.28181400	-2.00565800	-0.93177200
H	0.83870000	-2.91971000	-1.29843200
N	0.48875200	-0.88702000	-0.64111700
C	-2.89486100	0.32401700	-0.65825700
O	-1.47692300	0.33190100	-1.03038000
C	-2.97614800	-0.45606600	0.67133900
C	-0.92779500	-0.88979600	-0.55688600
C	-1.52416900	-0.95009600	0.93141600
H	-3.30907900	0.17826700	1.50089500
H	-0.91835900	-0.22086000	1.49611000
O	-1.41249300	-2.17824100	1.43564800
H	2.06745200	3.01730300	0.94747900
O	-3.84534900	-1.53823400	0.46222500
H	-3.63970700	-2.23021800	1.10745100
C	-3.37524700	1.75716300	-0.65843900
H	-4.37356400	1.79953000	-0.21902900
H	-3.41201300	2.15481500	-1.67560400
O	-2.43703100	2.53378300	0.12682600
H	-2.84381200	3.35061500	0.43443800
H	-3.44988500	-0.24308400	-1.40960600
H	-1.31937200	-1.74026400	-1.11765000
Li	-0.66406300	2.00096700	-0.29215000

Li-N3-O′-O5′-C2H-2

C	2.17061000	1.98833300	0.69561900
N	3.42208600	1.56570500	0.69657700
C	3.70627800	0.32944900	0.22685900
C	2.63735900	-0.47366400	-0.22771600
N	1.06871400	1.32359400	0.27994400
C	1.36591600	0.08111500	-0.15452700
N	4.97669400	-0.08041100	0.21257900
H	5.21532100	-0.99965000	-0.12540100
H	5.69912700	0.53329300	0.55678600
N	2.59164500	-1.74992300	-0.75648400
C	1.33245900	-1.96853100	-0.99838200
H	0.91017800	-2.87654100	-1.40237500
N	0.51501100	-0.88444700	-0.66211300
C	-2.86226900	0.31023800	-0.70227900
O	-1.45860700	0.33574100	-1.04326300
C	-2.90581300	-0.41098200	0.68358600
C	-0.91407100	-0.92279800	-0.58516900
C	-1.50868200	-1.09341200	0.82859000
H	-3.03936100	0.34249200	1.48514700
H	-0.88286500	-0.54211800	1.53874000
O	-1.57681000	-2.43448000	1.20031500
H	-2.50555200	-2.67378700	1.34319000
O	-3.95802800	-1.24787900	0.81446300
H	2.00585100	2.99335000	1.07078400
C	-3.39014900	1.72516400	-0.73623300
H	-4.39679000	1.74711300	-0.31328100
H	-3.41992400	2.10211300	-1.76148500
O	-2.48257400	2.53154800	0.05221700
H	-2.89655700	3.36432900	0.30321800
H	-3.40676600	-0.29955000	-1.42937000
H	-1.26502400	-1.73563000	-1.22725300
Li	-0.67278900	1.98468200	-0.26805500

Li-N3-O′-O5′-C2H-3

C	2.54478400	1.95899200	-0.41185400
N	3.71485300	1.41614000	-0.12147200
C	3.77603200	0.09537100	0.15255100
C	2.57893000	-0.65307600	0.10746800
N	1.33620800	1.35948100	-0.47789300
C	1.41124100	0.03545300	-0.21372300
N	4.95768000	-0.45171000	0.45042500
H	5.03155100	-1.43756900	0.64668900
H	5.78391300	0.12640700	0.45302500
N	2.32804400	-1.99285400	0.31827700
C	1.04470900	-2.12555500	0.13509000
N	0.40695800	-0.92067900	-0.16903700
C	-2.30113200	0.66838000	0.66127000
O	-1.39080700	0.55578900	-0.47511300
C	-3.09261200	-0.62545500	0.62571900
C	-0.96889400	-0.78029000	-0.64227100
C	-1.99006900	-1.63982600	0.17841900
H	-3.49038000	-0.88712100	1.61134700
H	-1.47972200	-2.02934200	1.08229700
O	-2.44783500	-2.73932500	-0.45985600
H	2.55663200	3.02382800	-0.62279000
O	-4.10707500	-0.51831800	-0.34247000
H	-4.52603400	-1.37958000	-0.46413500
C	-3.04283300	1.96931500	0.45830500
H	-3.61874000	2.21988500	1.35105700
H	-3.69989600	1.90450700	-0.40867100
O	-2.00630300	2.96126900	0.23002600
H	-2.37437900	3.84871400	0.29462700
H	-1.02626400	-1.02316000	-1.70399200
H	0.49866500	-3.05574900	0.18541200
H	-1.70135200	0.71467200	1.57793000
Li	-0.43498600	2.20222800	-0.64381600

International Journal of
Molecular Sciences

Na-N3-O′-O5′-C2H-1

C	2.23302500	1.78863000	0.99789200
N	3.49718300	1.41633000	0.88853800
C	3.79779300	0.28041900	0.21882200
C	2.73387200	-0.47539100	-0.31954100
N	1.13384900	1.16658500	0.51374300
C	1.45096000	0.01970100	-0.11679000
N	5.07810800	-0.08010700	0.09708400
H	5.32802600	-0.93040900	-0.38312100
H	5.79525700	0.48941600	0.51927400
N	2.70264800	-1.66650700	-1.02165600
C	1.44159500	-1.89665400	-1.23498400
H	1.02956300	-2.75395300	-1.74670600
N	0.60472400	-0.90067200	-0.71666400
C	-2.88023800	-0.05117500	-0.62473500
O	-1.49767800	0.13001500	-1.05337400
C	-2.79841600	-0.62901300	0.80192800
C	-0.80784400	-1.02175400	-0.57334100
C	-1.33175900	-1.13490500	0.90857800
H	-2.99400600	0.14315400	1.55464600
H	-0.69885000	-0.43622800	1.48998600
O	-1.16625700	-2.35583800	1.44278300
H	2.05596200	2.71185500	1.54121600
O	-3.72551200	-1.68056200	0.89662900
H	-3.43236700	-2.29462300	1.58435100
C	-3.63558000	1.24332200	-0.79997900
H	-4.65231500	1.09524400	-0.42927300
H	-3.67956500	1.51971900	-1.85712800
O	-2.97129400	2.28931500	-0.05504000
H	-3.60951000	2.96397200	0.19720500
H	-3.34749000	-0.81045000	-1.26051100
H	-1.12517900	-1.90858900	-1.12855400
Na	-0.73814800	2.25990600	-0.34579700

Na-N3-O′-O5′-C2H-2

C	-2.62874000	1.98088800	0.38004400
N	-3.82604300	1.43565800	0.23038600
C	-3.91524000	0.11123500	-0.01262700
C	-2.71982200	-0.63633900	-0.08303300
N	-1.41985400	1.38449600	0.32052400
C	-1.52569500	0.05929100	0.09515500
N	-5.12239200	-0.44183300	-0.17219100
H	-5.21425900	-1.43092500	-0.34167500
H	-5.94512500	0.13561100	-0.09527100
N	-2.49194400	-1.98304100	-0.28488300
C	-1.19980400	-2.11905000	-0.22898800
N	-0.53231800	-0.90544300	-0.01724400
C	2.73936200	0.31694700	-0.67891100
O	1.35041800	0.45423500	-0.25002300
C	3.14279300	-1.10821400	-0.30256500
C	0.86420800	-0.75448700	0.29805200
C	1.77743500	-1.85970300	-0.37750700
H	3.85379200	-1.53460400	-1.01725600
H	1.42992700	-1.97047400	-1.41657000
O	1.73061200	-2.99887100	0.32854300
H	-2.62263500	3.04948600	0.57491500
O	3.64078000	-1.12195900	1.01087700
H	3.67051500	-2.03591200	1.32485500
C	3.55644500	1.41700900	-0.03008400
H	4.56036500	1.42468000	-0.46084500
H	3.62733200	1.25824700	1.04602600
O	2.87674600	2.66584400	-0.30505700
H	3.51036300	3.38925200	-0.26296900
H	1.02978300	-0.77763100	1.37736100
Na	0.58422000	2.56601200	0.01701500
H	-0.65160800	-3.04492700	-0.31931700
H	2.73888100	0.44274200	-1.76448900

Na-N3-O′-C2H-1

C	-2.98166000	1.76949600	-0.13683000
N	-4.07745800	1.09623200	0.18193900
C	-4.02085100	-0.24991200	0.28014200
C	-2.78960700	-0.88546400	0.01571300
N	-1.75140300	1.28385300	-0.40368000
C	-1.72603200	-0.05898600	-0.32847000
N	-5.12668300	-0.92145400	0.62311300
H	-5.11342000	-1.92577000	0.70081000
H	-5.97894900	-0.41274600	0.79849800
N	-2.39770200	-2.21295100	0.04091600
C	-1.13938300	-2.20364900	-0.28807800
N	-0.65106100	-0.90927500	-0.53627900
C	2.94966400	-0.59237000	-0.69521900
O	1.64703300	-1.09355000	-0.34776500
C	3.14187600	0.82890400	-0.10321800
C	0.60597700	-0.50727800	-0.99020800
C	2.55611100	0.91711900	1.30577700
H	4.21478400	1.03939800	-0.03290000
H	3.06582200	0.31988500	2.07077700
O	1.58206100	1.59215900	1.54838300
H	-3.09008200	2.84867900	-0.18618400
O	2.48646700	1.81960900	-0.90506200
H	3.06978100	2.09802500	-1.61857200
C	3.98832600	-1.59745200	-0.23050800
H	4.94118900	-1.35636800	-0.71883500
H	3.67170900	-2.59254100	-0.55772900
O	4.10182700	-1.51729400	1.18215900
H	4.63136100	-2.24929500	1.51385700
H	3.01976900	-0.51188100	-1.78692300
H	0.73642300	-0.22218900	-2.03162300
Na	0.30082000	2.43119900	-0.17409600
H	-0.49288800	-3.06305200	-0.37450400
